# Complete Genome Sequence of Mumps Virus Genotype G from a Vaccinated Child in Franceville, Southeastern Gabon, in 2013

**DOI:** 10.1128/genomeA.00972-14

**Published:** 2014-11-26

**Authors:** Gael D. Maganga, Berthe A. Iroungou, Christine Bole-Feysot, Eric M. Leroy, Fousseyni S. Touré Ndouo, Nicolas Berthet

**Affiliations:** a Centre International de Recherches Médicales de Franceville (CIRMF), Département Zoonose et Maladies Emergentes, Franceville, Gabon; b CIRMF, Unité de Recherche et d’Analyse Médicale, Franceville, Gabon; c Aix Marseille University, URMITE unit, Marseille, France; d Imagine, Institut des Maladies Génétiques - Plateforme Génomique, Hôpital Necker, Enfants Malades, Paris, France; e Institut de Recherche pour le Développement (IRD), UMR 224, Laboratoire Maladies Infectieuses et Vecteurs, Ecologie, Génétique, Evolution et Contrôle (MIVEGEC), IRD/CNRS/UM1/UM2, Montpellier, France; f Centre National de la Recherche Scientifique, UMR 3569, Paris, France

## Abstract

The genome of mumps virus (MuV), a member of the family *Paramyxoviridae* of the genus *Rubulavirus*, consists of a single-stranded, negative-sense, nonsegmented RNA. Here, we report the first whole-genome sequence of 15,263 nucleotides of a mumps virus strain from a 6-year-old vaccinated boy in Franceville, southeastern Gabon.

## GENOME ANNOUNCEMENT

Mumps virus is a common and highly contagious viral disease characterized mainly by fever and parotitis. Although mumps virus infections are benign and usually not fatal, they can cause some complications, including aseptic meningitis, orchitis, pancreatitis, encephalitis, epididymitis, and deafness (1). The mumps virus (MuV) (order *Mononegavirales*, family *Paramyxoviridae*, genus *Rubulavirus*) genome contains genes encoding seven proteins: nucleocapsid (N), phosphoprotein (P), matrix (M), fusion (F), small hydrophobic (SH), hemagglutinin-neuraminidase (HN), and large (L) proteins. Based on a sequential analysis of the gene encoding the SH protein, 12 distinct genotypes (A to L) of MuV have been recognized by the World Health Organization (WHO) (2). Through vaccination, the disease had almost entirely disappeared, even if it was observed that MuV cases can occur in highly vaccinated populations (3–5). In 2000 and 2010, 934 cases and 1 case of mumps virus, respectively, were reported in Gabon (6). Here, we report the first whole-genome sequence of a MuV strain from a 6-year-old boy in Franceville, southeastern Gabon, in 2013. Viral RNA was extracted from a serum sample on a BioRobot EZ1 automat (Qiagen) using the EZ1 Virus minikit version 2.0, according to the manufacturer’s instructions. RNA was retrotranscribed using SuperScript III enzyme prior to amplification using the Phi29 enzyme, as described previously (7). A barcoded library was prepared from 500 ng of amplified DNA using the Ion Xpress Plus genome DNA (gDNA) and Amplicon library preparation kit. Sequencing was performed on an Ion 316 chip using the Ion PGM 300 sequencing kit, as recommended by the manufacturer. A total of 5,034,817 reads were obtained and were filtered according to quality, and those corresponding to the human genome sequence were filtered by mapping on the *Homo sapiens* hg 19 sequence using the Bow tie 2.0 software. The viral reads corresponding to the mumps virus genome were selected and assembled using ABySS and CAP3 in order to obtain the full-length genome. Our MuV genome is composed of 15,263 nucleotides, and genomic analysis showed the classical organization of *Paramyxoviridae*, with seven transcription units containing seven genes and two noncoding regions located at the 5′ and 3′ ends of the genome. Although many cases of mumps virus in several African countries were reported to the WHO, the circulating genotypes were rarely known. The genotype of this Gabonese MuV strain was determined based on a phylogenetic analysis of the partial sequence of 316 nucleotides of the SH gene compared to the sequences available in the databases. Our strain has 97% amino acid identity with sequences belonging to genotype G strains and shows the highest amino acid identity with strain accession no. EU606261 circulating in the United Kingdom in 2005. In these cases reported in Franceville, MuV was detected mainly in children who had received two doses of the measles-mumps-rubella vaccine. The introduction of MuV genotype G in Gabon is due to a decrease in immunization coverage in children <1 year old based on the data available for the measles virus vaccine (8).

### Nucleotide sequence accession number.

This whole-genome shotgun project has been deposited in DDBJ/ENA GenBank under the accession no. KM597072.
